# Effects of animated pedagogical agent-guided loving-kindness meditation on flight attendants’ spirituality, mindfulness, subjective wellbeing, and social presence

**DOI:** 10.3389/fpsyg.2022.894220

**Published:** 2022-08-03

**Authors:** Chao Liu, Hao Chen, Fang Zhou, Chao-Hung Chiang, Yi-Lang Chen, Kan Wu, Ding-Hau Huang, Chia-Yih Liu, Wen-Ko Chiou

**Affiliations:** ^1^School of Journalism and Communication, Huaqiao University, Xiamen, China; ^2^Business Analytics Research Center, Chang Gung University, Taoyuan, Taiwan; ^3^School of Film Television and Communication, Xiamen University of Technology, Xiamen, China; ^4^Department of Economic and Management, Suzhou Vocational Institute of Industrial Technology, Suzhou, China; ^5^Department of Shipping and Transportation Management, National Penghu University of Science and Technology, Magong, Taiwan; ^6^Department of Industrial Engineering and Management, Ming Chi University of Technology, New Taipei, Taiwan; ^7^Department of Orthopaedic Surgery, Chang Gung Memorial Hospital, Taoyuan, Taiwan; ^8^Institute of Creative Design and Management, National Taipei University of Business, Taoyuan, Taiwan; ^9^Department of Psychiatry, Chang Gung Memorial Hospital, Taoyuan, Taiwan; ^10^Department of Industrial Design, Chang Gung University, Taoyuan, Taiwan

**Keywords:** animation guidance, loving-kindness meditation, mindfulness, spirituality, subjective wellbeing

## Abstract

Loving-kindness meditation (LKM) was first practiced by Buddhists and then developed by clinical psychologist. Previous studies on LKM have mainly focused on the impact of real person-guided meditation on depression, anxiety, and other negative psychology. During the COVID-19 pandemic, this study explored the effect and mechanism of media-guided LKM on the improvement of social presence, mindfulness, spirituality, and subjective wellbeing (SWB). From the viewpoint of positive psychology, this study compared the different media effects of animated pedagogical agent (APA)-guided LKM and audio-guided LKM. A total of 82 flight attendants were recruited from airlines; then, they were randomly assigned to two groups: APA group (41 participants) and audio group (41 participants), which both underwent an 8-week LKM training intervention. The aforementioned four main variables were measured pre and post the meditation experiment. The results indicated that both APA-guided meditation and audio-guided meditation significantly improved subjects’ spirituality and SWB. Compared with audio-guided meditation, APA-guided meditation significantly improved the subjects’ spirituality, SWB, and social presence. Audio-guided meditation has no significant effect on social presence. This study highlights APA-guided meditation has a positive effect on spirituality, SWB, and social presence, which may provide individuals with a simple and easy method to improve their mental health.

## Introduction

The COVID-19 outbreak is considered to be the seminal event and major health crisis in the history of the world today (Sachs et al., 2020). Airline cabins are public places that gather many people, service is accomplished through interaction between staff and guests, and some airlines have seen outbreaks and cases of COVID-19 (Pombal et al., 2020). In addition, many airlines experienced severe layoffs during the pandemic, and the COVID-19 pandemic has had a significant impact on the mental health of flight attendants (Görlich and Stadelmann, 2020). On the one hand, flight attendants shape the image of the airline, and on the other hand, they are also related to aviation safety, so the mental health of flight attendants is very important (Grout and Leggat, 2021). Previous research has found that the quality of vertical communication implemented by airline management can reduce job insecurity among flight attendants during the COVID-19 pandemic in 2019 (Charoensukmongkol and Suthatorn, 2021). In addition to alleviating flight attendants’ anxiety, depression, and other negative emotions, loving-kindness meditation (LKM) training was found to improve flight attendants’ subjective wellbeing (SWB). Due to the impact of the epidemic, offline activities were increasingly restricted. In the post-epidemic era, many offline activities in the past have turned to online alternatives (Onderdijk et al., 2021), and online learning has become an important way of remote education. Online meditation training overcomes the limitations of time and space in traditional meditation training, making learning more flexible and convenient (Cavalera et al., 2019). Students can practice meditation at anytime and anywhere, which is more in line with the busy life pace of flight attendants.

However, due to the nature of remote education that the teaching and learning are separated in time and space, online learning is mostly realized in the virtual learning environment of human–computer interaction, and there is a lack of face-to-face communication between learners and teachers (Heradio et al., 2016). In the process of human–computer interaction, some real social clues are virtually filtered out, leading to the decrease of online communication emotions, thus reducing the learning effect (Themeli and Bougia, 2016). Some studies show that social presence can promote social interaction in virtual learning environment, enhance online learners’ sense of interaction, and improve learning effect (Cheng and Wang, 2019). In virtual learning environment, social presence is an important concept to describe how to conduct virtual social interaction. Social presence was regarded as an attribute of media, and the degree of social presence varies with different media (Short et al., 1976). Therefore, this study aims to compare audio and animation to find out which media form is more suitable for the online training of LKM for flight attendants and remotely intervene their mental health to improve their SWB.

### Loving-kindness meditation, mindfulness, spirituality, and subjective wellbeing

Loving-kindness meditation is a special meditation to cultivate compassion (Chen et al., 2021), and this kind of compassion is a kind of unconditional and undifferentiated goodwill to all living beings, that is, this goodwill is not affected by kinship or interest relations (Liu et al., 2022). The main function of LKM is that it can be practiced to regulate real-time emotions in life when the mood is low, and long-term practice of LKM is conducive to long-term emotion regulation. LKM can help practitioners counter negative experiences of stress, pain, and mental illness (Zeng et al., 2019).

Mindfulness is about being aware in a certain way, purposefully aware, living in the moment, and without mental judgment. Mindfulness was negatively correlated with emotional exhaustion among call center workers in the Philippines (Charoensukmongkol and Puyod, 2022), anxiety in English speech class of College students in Thailand (Charoensukmongkol, 2019), and perceived stress among Thai flight attendants (Suthatorn and Charoensukmongkol, 2022) and was positively correlated with the cross-cultural sales performance of Thai international trade fair salespeople (Charoensukmongkol and Pandey, 2021). Both LKM and mindfulness are derived from Buddhism and emphasize the focus on the present, and both can improve emotional wellbeing, so some studies suggest that there is a certain correlation between the two (Fredrickson et al., 2017).

Spirituality is a person’s pursuit and experience of connection with the essence of life. It includes three dimensions: connection to oneself, connection to others and nature, and connection to transcendental experience. Previous research has explored the antecedent variables of spirituality and found that LKM can facilitate personal spiritual growth by moving away from a preoccupation with the actions and reactions of the self and enhancing a connection to the universal human experience of altruism, love, and compassion (Kristeller and Johnson, 2005; Liu et al., 2020b). LKM can internally enhance the unified inner connection of the individual and externally enhance altruism, transcend egocentrism, and feel a sense of energetic connection to other beings in the universe (McClintock et al., 2016).

Subjective wellbeing consists of two parts, namely, cognitive component and affective component (Diener et al., 2018). Cognitive component refers to life satisfaction, which is an individual’s overall summary, cognition, and evaluation of his life and is a key indicator of SWB. The cognitive component is a more effective positive measure, independent of the affective component (Moore and Diener, 2019). Emotional components are divided into positive and negative emotions, which are relatively independent and subordinate to SWB (Jebb et al., 2020). Previous studies have found that SWB is positively correlated with mindfulness and spirituality (Liu et al., 2020a), and LKM can improve the mindfulness, spirituality, and SWB of flight attendants (Liu et al., 2020b).

### Social presence

The theory of social presence was first proposed by Short et al. (1976) that social presence refers to the degree to which a person is regarded as a “real person” and the perceived degree of connection with others in the process of communication through media. In short, social presence is the ability to connect or perceive media and is regarded as an attribute of media. Gunawardena and Zittle (1997) suggested that social presence was not only an attribute of media, but also a psychological perception generated in the process of human interaction with media. Garrison et al. (1999) defined social presence as the ability of participants to attempt to project themselves socially and emotionally as real people through the use of communication media. Rogers and Lea (2005) argued that social presence is the sense of immersion caused by learners’ perception of belonging and identity in online learning groups, and the sense of “being on the scene” of users in virtual or intermediary environments. Biocca et al. (2003) suggested that social presence is “the feeling of being with others” in a virtual environment. The “others” mentioned here include other humans and other anthropomorphic forms of expression (e.g., agent and avatar) presented by technological media (e.g., text, image, video, and animation). This sense of social presence is not the existence of physical facts, but the existence of psychological perception (Biocca et al., 2003). The different viewpoints of the above scholars are only from different angles to look at social presence, and they are not opposed to each other. In a word, social presence is not only a media attribute, but also a psychological perception generated by users when they communicate with media (Triberti et al., 2018).

### Animated pedagogical agent

Face-to-face communication was thought to provide the highest level of social presence, while computer-mediated communication (such as email) was once thought to be associated with lower levels of social presence due to the reduced ability of this medium to convey social cues (e.g., facial expressions, gestures, and sounds) (Short et al., 1976). With the rapid development of digital media technology, diversified media content provides more and more abundant social clues and improves the sense of social presence of computer media. In social presence theory, teaching is understood as a communicative behavior, that is, a social activity that depends on the social partnership between teachers and learners (Mayer and DaPra, 2012), and social interaction is an important mechanism of teaching and learning activities (Chin et al., 2016). Traditional classroom teaching involves face-to-face learning, with teachers explaining important knowledge and often using social cues such as gestures and expressions to guide attention and help students understand the content (Wang et al., 2018). Animated pedagogical agent (APA) is a cartoon character implanted in multi-media materials. APA can not only convey some social cues through humanoid features, but also use anthropogenic non-verbal behaviors (e.g., gestures, body movements, and facial expressions) in network situations as social cues to promote learning (Chin et al., 2016). These APAs have a positive impact on factors such as learner attention, perception of material, and learning performance (Mayer and DaPra, 2012). Thus, when an APA is integrated into a multi-media environment, it increases the learner’s social response and promotes their interest in learning tasks (Chin et al., 2011). In addition, these agents are able to establish a simulated person-to-person social interaction to simulate learner–teacher communication (Hong et al., 2014). APAs act as personal mentors and advise learners to enjoy and stay in a computer-based learning environment to enhance their sense of social presence (Hong et al., 2014).

Many previous studies have found that adding APA in a multi-media environment can promote users’ learning of multi-media courses, promote learners to process materials more actively, and form better learning results (Yung and Paas, 2015). Chin et al. (2016) found that adding APA into the computer-aided learning system can successfully improve students’ learning interest and motivation. In virtual learning environments, the enhanced social presence of learners’ perception of APA can effectively improve their learning outcomes (Wang et al., 2018). Previous studies on the impact of APA on students’ learning performance in multi-media teaching found that learners in the APA group had better learning performance than those without the APA group (Mayer and DaPra, 2012). Moreno et al. (2001) found that learners in an environment with APA had better learning performance than in an environment with only text and image resources. Studies on the influence of APA on learners’ subjective feelings show that in multi-media learning, APA can significantly reduce learners’ perception of task difficulty, stimulate their interest in learning, enhance their confidence in learning, and make it easier for learners to conduct meaningful learning (Chin et al., 2011). In addition, studies exploring the influence of APA on the distribution of learners’ attention and meaning found that communication between learners and APA was similar to interpersonal communication in real life (de Back et al., 2021). The use of APA in multi-media learning will not only affect learners’ attention allocation and subjective feelings, but also have a positive impact on their learning performance (Dunsworth and Atkinson, 2007).

### Research purposes and hypotheses

The mental health of flight attendants has been greatly affected during the COVID-19 pandemic. On the one hand, flight attendants shape the image of the airline, and on the other hand, they are closely related to aviation safety. During the epidemic, most offline group meditation training institutions were closed, so online LKM training is a good choice. According to the theory of social presence, using cartoon images as virtual meditation instructors, namely adding APA to LKM online training, can attract participants’ attention and improve their interest in learning. At the same time, the interaction between APA and participants also contains rich social clues, which can improve participants’ social presence and thus improve the training effect.

There are few studies exploring influence of APA on the effect of online meditation training, and this study attempted to fill this gap. Therefore, the purpose of this study was to compare the different effects of online LKM training between participants in the APA-guided LKM meditation group (APA group) and participants in the control group without APA-guided LKM training (audio group) and to examine whether APA-guided online LKM training can improve the social presence, mindfulness, spirituality, and subjective wellbeing of flight attendants.

Based on the above theories, we propose the following hypotheses:

Hypothesis 1. The post-test scores of social presence, mindfulness, spirituality, and subjective wellbeing in APA group were significantly higher than the pre-test scores.

Hypothesis 2. The post-test scores of social presence, mindfulness, spirituality, and subjective wellbeing in APA group were significantly higher than those in audio group.

## Methods

### Participants

The participants of this study are flight attendants working in an airline company in China. We have posted a recruitment advertisement for online LKM training on the internal network of a domestic airline, saying that LKM is a self-exploration activity that helps the practitioners to better understand themselves, release pressure, regulate emotions, and improve happiness. Flight attendants who were interested and qualified to participate in our LKM study provided their registration information. Finally, 82 eligible participants were recruited. The participants were divided into two groups: APA (*n* = 41)- and audio (*n* = 41)-guided meditation and strictly match the proportion of the gender and education levels, so the differences in demographic factors such as age composition and sex ratio composition between the two groups were not statistically significant (Table 1). The informed consent of each participant was obtained.

**TABLE 1:** Demographic characteristics of participants.

Characteristic		Total	APA	Audio
Age (SD)		28.48 (7.25)	27.64 (7.63)	29.32 (6.92)
Gender	Male (%)	32(39%)	16(39%)	16(39%)
	Female (%)	50(61%)	25(61%)	25(61%)
Education	High school degree (%)	7(8%)	4(10%)	3(7%)
	Associate degree (%)	44(54%)	22(54%)	22(54%)
	Bachelor degree (%)	31(38%)	15(36%)	16(39%)

### Instruments

Mindfulness Attention Awareness Scale (MAAS) is used to measure individuals’ trait mindfulness, which was developed by Brown and Ryan (Brown and Ryan, 2003) and was revised in Chinese by Deng et al. (2012). This scale is unidimensional structure, 15 items, six-point Likert scale, with “1” to “6” indicating “almost always” to “almost never.” The higher the score, the more the mindfulness. Of the many instruments that measure trait mindfulness, the MAAS is the most widely used (Ryan et al., 2021). Numerous studies have shown that the MAAS has good validity in culturally diverse populations with different experiences of mindfulness meditation (MacKillop and Anderson, 2007). In this study, Cronbach’s alpha was 0.92.

The Spiritual Attitude and Involvement List (SAIL) was developed by de Jager Meezenbroek et al. (2012). The scale is based on the consideration that spirituality is a universal human experience and is appropriate for measuring the level of spirituality in people of different religious and non-religious faiths. It has 26 items and six dimensions (including meaning, belief, acceptance, concern for others, connection to nature, and spiritual involvement dimensions). A six-point Likert scale is used, ranging from 1 to 6 on a scale of “not at all” to “very well,” with higher scores indicating higher levels of spirituality (de Jager Meezenbroek et al., 2012). The scale has been translated into several languages and used in many countries and has shown good validity (Jirasek and Hurych, 2018). In this study, Cronbach’s alpha was 0.91.

Subjective wellbeing uses the Satisfaction with Life Scale (SWLS) and the Positive and Negative Affect Scale (PANAS) to measure SWB. The SWLS was developed by Diener, Emmons, Larsem, and Griffin (Diener et al., 1985), and its Chinese version was revised by Wu and Yao (Wu and Yao, 2006). The scale has five items and a seven-point Likert scale ranging from “completely disagree” to “strongly agree.” The higher the score, the more satisfied with your life. In this study, Cronbach’s alpha was 0.89. Positive and Negative Affect Scale (PANAS) was developed by Watson, Clark, and Tellegen (Watson et al., 1988), and its Chinese version was revised by Sheldon (Sheldon et al., 2004). The scale consisted of 20 items, a five-point Likert scale ranging from “none at all” to “all the time.” Higher scores indicate more frequent occurrence of the corresponding emotion. In this study, Cronbach’s alpha was 0.88. The standard score for life satisfaction was added to the standard score for positive emotions and subtracted from the standard score for negative emotions as the score for subjective wellbeing (Diener et al., 1985).

Social Presence Scale (SPS) measures the social presence of a medium and was first developed by Gunawardena and Zittle who divided social presence into three dimensions: emotional response, interaction response, and cohesive response. The scale has 17 questions and is scored on a five-point Likert scale: “5” indicates very tedious, and “1” indicates very stimulating (Gunawardena and Zittle, 1997). Previous studies have shown that the SPS has good validity (Song et al., 2021). The Chinese version of SPS was adapted by Zou et al. (2021) presenting the same structure. In this study, Cronbach’s alpha was 0.93.

This study chose SPS as the measuring tool of social presence, because Gunawardena and Zittle studied social presence under the background of online education and developed this scale (Gunawardena and Zittle, 1997), which is close to the research topic of this study. This study also focuses on online education and compares the different effects of animation and audio media on LKM online training effect and social presence. Previous studies have also used SPS to measure social presence in online nursing courses (Burruss et al., 2009).

### Loving-kindness meditation intervention

**FIGURE 1:**
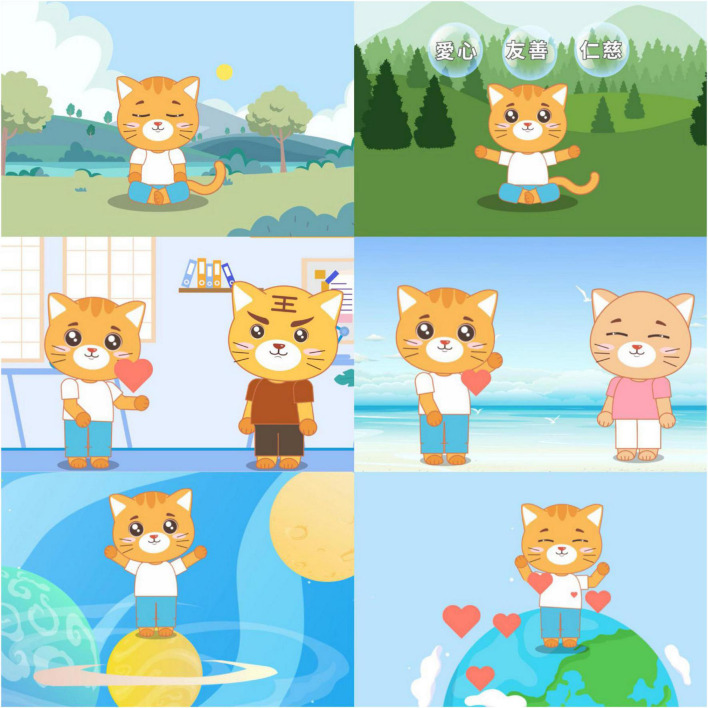
Animated pedagogical agent (APA) cartoon image design.

In this study, the image of APA was designed as a cute anthropomorphic cat (see Figure 1), because cats are generally regarded as gentle and lovely. At the same time, we designed different facial expressions and body movements for APA to cooperate with the explanation of different contents. The audio material of meditation instruction is voice explanation recording, which only contains simple voice narration and soothing music in the background. Animation materials for meditation instruction are composed of APA and audio material. The audio-guided materials and the APA-guided materials were exactly the same in the sound part. The only difference was that the animation-guided materials contained visual information such as APA cartoon images and animation, while the audio materials only contained auditory information without visual information.

The animation and audio production materials were adapted from previous research, and the production process included designing and drawing APA cartoon characters using drawing software (SAI). Then, animation software (Flash) was used to create interactive animations in SWF format with a resolution of 1,920 × 1,080 pixels. Finally, voice actors were invited to record narration materials. The animation and audio can be watched for 5 min at a time and can be repeated in a loop. APA-guided meditation animation and audio were eventually uploaded to the website to facilitate monitoring and assessment of each user’s viewing time and meditation practice time per day. Using these data, the researchers calculated and assessed whether the 8-week period of effective intervention was sufficient. Meditation intervention materials are designed to provide online training of LKM intervention for flight attendants during the epidemic, mainly providing animation and audio guidance for flight attendants to learn LKM.

The meditation animation and audio introduce methods of compassion meditation, which include putting yourself in a comfortable position first to prepare for blessings and then spreading blessings to that object in four ways: (1) may you have no enemies; (2) may you have no pain; (3) may you have no disease; (4) may you have your own happiness. The order of the objects of blessing is as follows: (1) bless oneself; (2) bless the loved ones; (3) bless neutral people, that is, people who do not like or dislike them; (4) bless those whom you hate; (5) bless all people or all living beings.

### Procedure and design

We advertised on an airline intranet and flight attendants who were interested and eligible to participate in our LKM study provided their registration information. Inclusion criteria were as follows: (1) adults aged 18 years or older; (2) able to speak and read in Chinese sufficiently to complete the questionnaire; (3) worked as a flight attendant for at least 1 year. Exclusion criteria were as follows: (1) self-reported depression, anxiety, bipolar disorder, substance abuse, or suicide; (2) prior experience with LKM or mindfulness meditation.

In this study, a two-arm randomized controlled trial was conducted. The intervention condition was APA-guided LKM exercise, and the control condition was audio-guided LKM exercise. A 2 (group) × 2 (time) parallel experimental design was used in this study. Group members were assigned to groups using random sequence codes generated by SPASS 22 software. These code lists, initially labeled “Group A” and “Group B,” were provided to participants who were randomly assigned to either group A or group B using A 1:1 allocation ratio and strictly match the proportion of the gender and education levels. The interveners were not given a grouping list during the intervention. After the data analysis was completed, the group members informed the data analyst and the experimental interventionist of the meaning of each group in the group list. Double blindness is realized by this program.

We randomly assigned 82 eligible participants to either animation intervention group (41 participants) or the audio meditation intervention group (41 participants). During the experiment, members of the two groups flew on separate flights without any contact. To maintain engagement and control attrition, there was a pre- and post-experiment trial fee of RMB 50 per participant at the beginning of the survey to increase their motivation. If the animated meditation or audio meditation was used for more than half an hour per day during the 8-week meditation intervention (the amount of time each user spent using the animation and audio per day was visible through the video monitoring software), a test fee of RMB 100 was given at the end of the 8-week intervention. Those who drop out or fail to meet the daily usage time within the 8 weeks will not receive the RMB 100 test fee.

**FIGURE 2:**
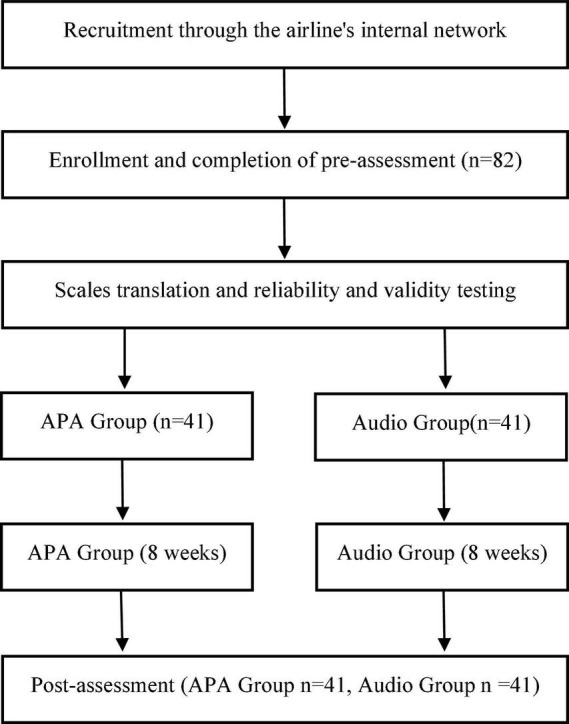
Procedure flowchart.

Participants complete an online consent form through a secure online survey platform, provide demographic information, and complete the following questionnaires (pre-test): (1) Mindfulness Attention Awareness Scale (MAAS); (2) Spiritual Attitudes and Involvement Inventory (SAIL); (3) Positive and Negative Affect Scale (PANAS); (4) Satisfaction with Life Scale (SWLS); (5) Social Presence Scale (SPS, Social Presence Scale is based on the text introduction to compassionate meditation as a baseline). It took approximately 20 min to answer the questionnaire. The APA group was given an animated meditation intervention 3 times a week for 8 weeks, and the audio group was given an audio meditation intervention. Both the two groups were invited to a 20-min zoom webinar which provided an overview of how to use the animated and audio meditations. All participants began the 8-week study on the Monday following the zoom webinar. At the end of the intervention period (week 8), participants completed the same questionnaire again (post-test) and were compensated with an additional RMB 100 (Figure 2). This study followed an online funnel reporting procedure. Participants were asked whether they knew the purpose of the study and the topic being investigated and whether they knew the same questions in the pre-test and post-test. To recruit subjects who were oblivious to the experimental conditions and naively used animated meditations, the funnel report helped to obtain a homogeneous sample in both groups. Participants were given the opportunity to record any number of questionnaires anonymously and could withdraw at any stage. The study was approved by the ethics committee of the Chang Gung University (IRB No: 201901236B0), and the protocol was carefully reviewed to ensure that it complied with the ethical guidelines of China Psychological Association.

### Data analysis

SPSS 22 software was used for statistical analysis of the data in this study. The confidence interval was set as 95%, and the significance level was set as 0.05. In this study, descriptive statistics were used to describe the distribution of basic data of subjects, and ANOVA was used to compare the differences pre- and post-intervention and between groups.

## Results

Four 2 (Group Type: APA, Audio) × 2 (Time: Pre, Post) ANOVA with repeated measures was conducted on mindfulness (MAAS), spirituality (SAIL), SWB, and social presence (SPS). To test for the effects of group type differences in how different guidance media influence the effect of LKM meditation, the two factors (gender and education) were controlled and excluded from the ANOVA. The *p*-values of Box’s test and Mauchly’s test were all greater than 0.05, which showed that the observed covariance matrices of the dependent variables are equal across groups, indicating that these data were suitable for ANOVA.

**FIGURE 3:**
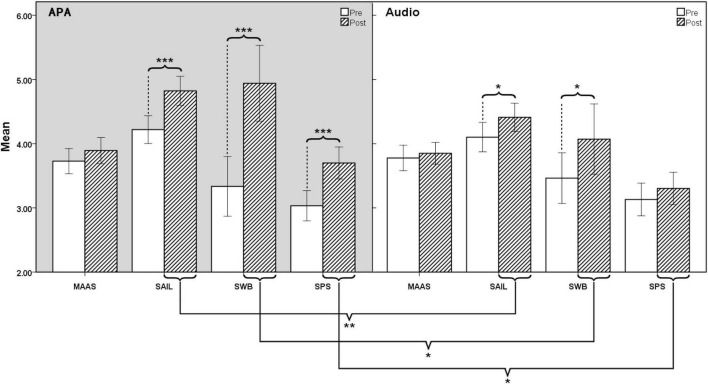
Comparison of four measures between animated pedagogical agent (APA) and audio group. *p < 0.05; **p < 0.01; ***p < 0.001.

The results of the ANOVA are presented in Table 2 and Figure 3, and the descriptive statistics are presented in Table 3. We found a significant main effect of time for spirituality, SWB, and social presence. There was a significant main effect of group for spirituality, and a significant interaction between time and group for SWB and social presence.

**TABLE 2:** ANOVA results.

Measure	Variable	F	p	η^2^
MAAS	Time	2.679	0.104	0.033
	Group	0.001	0.973	<0.001
	Time × Group	0.405	0.527	0.005
SAIL	Time***	26.750	<0.001	0.251
	Group*	4.197	0.044	0.050
	Time × Group	2.776	0.100	0.034
SWB	Time***	27.826	<0.001	0.258
	Group	1.696	0.196	0.021
	Time × Group*	5.655	0.020	0.066
SPS	Time***	13.210	<0.001	0.142
	Group	1.326	0.253	0.016
	Time × Group*	4.484	0.035	0.054

Only significant differences are marked with *; *p < 0.05; ***p < 0.001.

**TABLE 3:** Means and standard deviations for each measure of each group, pre- and post-assessment.

Group	Measure	Mean (SD)	
		
		Pre	Post
APA	MAAS	3.728 (0.623)	3.893 (0.648)
	SAIL	4.219 (0.687)	4.823 (0.719)
	SWB	3.336 (1.473)	4.941 (1.873)
	SPS	3.033 (0.749)	3.699 (0.789)
Audio	MAAS	3.778 (0.630)	3.851 (0.534)
	SAIL	4.102 (0.726)	4.411 (0.698)
	SWB	3.463 (1.252)	4.071 (1.740)
	SPS	3.131 (0.807)	3.303 (0.796)

## Discussion

### Why mindfulness is not significant

Neither the APA-guided LKM nor the audio-guided LKM improved the participants’ mindfulness. Mindfulness can generally be defined as non-judgmental awareness of the mind, body, and external environment in each present moment. Mindfulness consists of three key components:(1) non-judgmental; (2) live in the moment; (3) internal and external awareness. The practice of LKM begins with blessing oneself, spreads to friends, and eventually to strangers. From the object’s point of view, the object of LKM is rough and open (Komagata and Komagata, 2010). In practicing LKM, the multiple changes of objects in a short period of time can lead to disorganization of the mind, leading to a higher degree of inattention, which is definitely not good for the promotion of mindfulness, because the state of mindfulness requires clear internal and external awareness (Sheldon et al., 2015). In practicing LKM, the participants’ perception of the external environment was impaired not only because the object of the blessing required extreme concentration, but also because the internal perception was also affected by the repeated changes of the blessed object, which affected the participants’ mindfulness (Zeng et al., 2017).

### Animated pedagogical agent-guided loving-kindness meditation improves social presence

The results showed that APA-guided LKM training significantly improved participants’ social presence, while audio-guided LKM intervention had no significant effect on social presence of participants. The reason may be that the cartoon anthropomorphic image of APA induces automatic social responses in the subjects. Visual information of APA provides a means to be recognized and identified, which can enhance users’ sense of participation in the virtual environment (Nowak and Biocca, 2003). The idea is that people automatically react socially to entities that look or behave like humans. Thus, if an agent looks or acts like a person, people will react socially to it in the same way they would react to another person (Reeves and Nass, 1996). There is behavioral and neuropsychological evidence that humans are hardwired to respond to intentional social cues from entities and that human-like entities may get more special attention (Nowak and Biocca, 2003). APA in a virtual environment can activate selective neuropsychological responses that result in the user’s perception of these entities as “living” rather than “inanimate” (Gainotti et al., 1995). In other words, APA can activate people’s tendency to respond socially. This could mean that people are reluctant to distinguish between humans and non-humans in the face of APA with human-like morphology and behavior, because humans tend to be confused about what is real and perceived to be real, and people automatically use social rules to guide their behavior toward these media (Nowak and Biocca, 2003). Unlike physical environments, social communication in virtual environments may be based on limited social cues (Hess et al., 2009). APA can enable users to generate strong automatic social responses from fewer social cues and enhance social presence (Benford et al., 2001). The social cues APA conveys can trigger patterns of social dialog in users. Once users are aware that social interactions are taking place, they use the rules of human interaction to treat these interactions with APA as social partners (Mayer and DaPra, 2012). Even when users are well aware that APA is not a true mentor but merely a pedagogical agent, they still have a social reaction to APA (Dunsworth and Atkinson, 2007). Users have a social reaction to APA, and the social cues of animated characters increase the social presence (Di Natale et al., 2022). Thus, subjects in the APA group experienced a greater social presence than those in the audio group.

### Animated pedagogical agent-guided loving-kindness meditation improves spirituality and subjective wellbeing

It can be seen from the results that the spirituality and SWB of subjects were significantly improved by APA-guided LKM training, and the significance was higher in the APA group than in the audio group. The reason may be that APA helped improve the learning outcomes of the subjects and helped them to perform better LKM exercises, which led to more significant improvements in spiritual and SWB. Animation is more vivid than audio and conveys more social cues. The vividness of media was considered to influence users’ perception of social existence (Lombard and Ditton, 1997), because the increased vividness can provide additional social clues, allow users to better identify various social cues presented in human–computer interaction, and increase users’ perception of social presence (Moreno et al., 2001). Therefore, APA’s cartoon images are more vivid, which can increase users’ perception of social existence, while less vivid audio cannot fully convey key social clues (e.g., facial expressions, gestures, and body movements) due to the lack of important visual information and may lead to lower social existence. Daft and Lengel (1986) argued that the level of social presence was highly correlated with the number of social cues in the media. Social presence is the detection of specific cues in a virtual environment that may be related to the behavior of other entities (Triberti et al., 2018). In addition, social presence is not limited to media interaction, but is described as a basic psychological process that exists in real environments and daily life (Triberti et al., 2018). Given enough social cues, users can easily perceive their interactions with animated characters as social interactions. Bringing verbal (oral) and non-verbal social cues (e.g., facial expressions, gestures, and body movements) into a multi-media environment can simulate the interaction between people, thus promoting user’s participation in the learning process (Moreno et al., 2001). Once this simulated person-to-person connection is established, social communication between students and APA is considered natural and automatic, following the rules of human communication, just as in real person-to-person conversations (Dunsworth and Atkinson, 2007). Participants naturally engage in a subject-user relationship, just as they would interact with a tutor or teacher in a classroom (Lin et al., 2013). Thus, in this case, the user has an underlying motivation to understand the information presented to him/her and is more likely to process the information in depth to achieve meaningful learning (Lin et al., 2013). A lively pedagogical agent helps to improve learning performance and outcomes by maintaining the user’s attention and engaging the user in the cognitive process of teaching (Yung and Paas, 2015). APA can help users gain a deeper understanding of the topic they are learning, just as users learn from real teachers (Wang et al., 2018). To sum up, the APA convey social cues (e.g., expressions, gestures, and body movements) launched the user’s social interaction model (i.e., the APA as real interaction partners), which in turn leads to the participants more effort to understand the material being presented (i.e., the cognitive process of organization and integration), leading to better LKM learning outcomes (significantly improved spirituality and SWB).

### Advice for flight attendants

This study explored the impact of APA-guided LKM on flight attendants’ mindfulness, spirituality, SWB, and social presence and provided a reference for how to improve flight attendants’ subjective wellbeing in a simple and practical way in the post-epidemic era. Airlines can establish a more standardized, systematic, and operable online LKM intervention program to improve the stress resistance and self-regulation ability of flight attendants and their mental health, thus helping to improve the overall service level of airlines and the market competitiveness of air transport. Airline purser can include animation-guided LKM training in the flight process, such as LKM guided by animation for 10 min in each pre-flight preparation, to cultivate flight attendants’ subjective wellbeing. Alternatively, flight attendants could be guided to reassess their positive and negative emotions to improve their ability to regulate those emotions and thus improve their ability to communicate with passengers. After the flight, LKM guided by animation can be used to improve the negative emotions of airline service personnel, help flight attendants effectively adjust and reduce psychological pressure, and reduce work anxiety. Compassion meditation can stimulate more positive emotions. At the same time, the training of compassion meditation for flight attendants is not only beneficial to individual physical and mental health, but also helps them quickly adapt to the new environment and work and face the negative emotions caused by conflicts with passengers with a good attitude.

### Research limitations and future studies

Due to the limited human and material resources, funds, and time, this study still has the following limitations and deficiencies: (1) The analysis of mindfulness, spirituality, social presence, and SWB is conducted on a holistic scale without analyzing its subdimensions; (2) in terms of research tools, the measurement in this study was a self-reported scale, which was subjective in the self-report of the subjects, and the study lacked objective measures such as physiological indicators and behavior. SPS was used as a measure of social presence in this study. Since APA was introduced into online education research in this study, some scholars pointed out that SPS may not be suitable for measuring social presence generated by artificial entities. Therefore, a more suitable scale will be selected to evaluate the social presence generated by APA in future studies; (3) samples were taken from a single unit, limited by factors such as the number of personnel, research funds, and nature of work, so the sample size was small; (4) due to the particularity and nature of the sample working environment, whether the results of this study can be extended to other populations requires further research verification.

Based on the limitations of this study, we put forward the following prospects for future studies: (1) Future studies will further conduct in-depth analysis on the subscales and subdimensions of each variable to explore the interaction between each dimension; (2) in addition to the use of self-reporting scales, more physiological objective evaluation indicators (such as EEG) will be added in future studies. A measure more suitable for evaluating the social presence of artificial entities (such as APA) will also be selected; (3) future studies will further expand the sample size and scope and further verify the generalizability and universality of the current results in other populations.

In addition, the following suggestions are put forward for the practical application of the research results: (1) Future studies should carry out online positive psychological intervention for people from different industries; (2) future studies should combine objective measurements such as physiological indicators and behavior to further explore the effects of online positive psychological intervention on subjects’ positive psychology; (3) future mental health workers should develop different cartoon characters according to different types of meditation to compare the experimental effects when using animation to improve the positive psychology of subjects.

## Conclusion

This study draws the following conclusions. First, both APA-guided LKM and audio-guided LKM significantly improved subjects’ spiritual and SWB; second, APA-guided LKM significantly improved subjects’ social presence, but audio-guided LKM had no significant effect on social presence; Third, compared with audio-guided LKM, APA-guided LKM significantly improved the subjects’ spirituality and SWB. Finally, we contribute to the literature of LKM and provide a deeper understanding of APA-guided LKM by linking mindfulness, spirituality, and SWB. Compared to audio materials, APA-guided LKM exercises significantly improved subjects’ spirituality, SWB, and social presence and enhanced their meditative effects. APA-guided online meditation as a simple and easy way of meditation training also has a certain practice and promotion value.

## Data availability statement

The raw data supporting the conclusions of this article will be made available by the authors, without undue reservation.

## Ethics statement

The studies involving human participants were reviewed and approved by the Chang Gung University Ethics Committee (IRB No: 201901236B0). The patients/participants provided their written informed consent to participate in this study.

## Author contributions

HC contributed with data collection, finding interpretation, and manuscript preparation. CL contributed to the experimental design, data collection, statistical analysis, and finding interpretation. C-HC and Y-LC were in charge of data collection, helped to prepare the experimental sites, and assisted with data collection. FZ, KW, D-HH, and C-YL assisted in the data collection and evaluation of the findings, as well as conceiving the project and participating in its interpretation. W-KC was responsible for the study’s conception and design, as well as the interpretation of the findings and manuscript writing. All authors read and approved the final version and submitted for publication.
